# Optimizing Behavioral Paradigms to Facilitate Development of New Treatments for Anhedonia and Reward Processing Deficits in Schizophrenia and Major Depressive Disorder: Study Protocol

**DOI:** 10.3389/fpsyt.2020.536112

**Published:** 2020-11-05

**Authors:** Amy C. Bilderbeck, Andreea Raslescu, Dennis Hernaus, Anja Hayen, Daniel Umbricht, Darrel Pemberton, Jane Tiller, Birgitte Søgaard, Anke Sambeth, Therese van Amelsvoort, Andreas Reif, Georgios Papazisis, Victor Pérez, Matilde Elices, Damien Maurice, Valérie Bertaina-Anglade, Gerard R. Dawson, Stephane Pollentier

**Affiliations:** ^1^P1vital Ltd, Wallingford, United Kingdom; ^2^School for Mental Health and Neuroscience, Maastricht University, Maastricht, Netherlands; ^3^F. Hoffmann-La Roche Ltd, Basel, Switzerland; ^4^Janssen Pharmaceutica, Beerse, Belgium; ^5^BlackThorn Therapeutics, San Francisco, CA, United States; ^6^H. Lundbeck, Valby, Denmark; ^7^Department of Psychiatry, Psychosomatic Medicine and Psychotherapy, University Hospital Frankfurt, Frankfurt, Germany; ^8^Department of Clinical Pharmacology, School of Medicine, Aristotle University of Thessaloniki, Thessaloniki, Greece; ^9^Institut de Neuropsiquiatria i Addiccions, Parc de Salut Mar, Barcelona, Spain; ^10^Hospital del Mar Medical Research Institute (IMIM), Barcelona, Spain; ^11^Departament de Psiquitria i Medicina Legal, Universitat Autònoma de Barcelona, Barcelona, Spain; ^12^Centro de Investigación Biomédica en Red de Salud Mental (CIBERSAM), Madrid, Spain; ^13^Biotrial Neuroscience, Rennes and Didenheim, France; ^14^Boehringer Ingelheim International GmbH, Ingelheim, Germany

**Keywords:** anhedonia, impaired motivation, negative symptoms, reward processing, reward deficits, schizophrenia, depression

## Abstract

**Background:** Behavioral tasks focusing on different subdomains of reward processing may provide more objective and quantifiable measures of anhedonia and impaired motivation compared with clinical scales. Typically, single tasks are used in relatively small studies to compare cases and controls in one indication, but they are rarely included in larger multisite trials. This is due to limited systematic standardization as well as the challenges of deployment in international studies and stringent adherence to the high regulatory requirements for data integrity. The Reward Task Optimization Consortium (RTOC) was formed to facilitate operational implementation of reward processing tasks, making them suitable for use in future large-scale, international, multisite drug development studies across multiple indications. The RTOC clinical study aims to conduct initial optimization of a set of tasks in patients with major depressive disorder (MDD) or schizophrenia (SZ).

**Methods:** We will conduct a multicenter study across four EU countries. Participants (MDD = 37, SZ = 37, with ≤80 age- and gender-matched healthy volunteers) will attend a study visit comprising screening, self-report and clinically rated assessments of anhedonia and symptom severity, and three reward processing tasks; specifically, the Grip Strength Effort task, the Doors task, and the Reinforcement Learning Working Memory task. The Grip Strength Effort and Doors tasks include simultaneous electroencephalography/event-related potential recordings. Outcomes will be compared using a two-way group design of MDD and SZ with matched controls, respectively. Further analyses will include anhedonia assessment scores as covariates. Planned analyses will assess whether our findings replicate previously published data, and multisite deployment will be evaluated through assessments of quality and conduct. A subset of participants will complete a second visit, to assess test–retest reliability of the task battery.

**Discussion:** This study will evaluate the operational deployment of three reward processing tasks to the regulatory standards required for use in drug development trials. We will explore the potential of these tasks to differentiate patients from controls and to provide a quantitative marker of anhedonia and/or impaired motivation, establishing their usefulness as endpoints in multisite clinical trials. This study should demonstrate where multifaceted reward deficits are similar or divergent across patient populations.

**Registration**: ClinicalTrials.gov (NCT04024371).

## Introduction

Anhedonia, a diminished ability to experience pleasure, is a symptom observed trans-diagnostically among individuals with central nervous system disorders (1). In clinical practice, anhedonia is closely linked to impaired motivation, loss of interest, apathy, and social withdrawal, which together predict poor functional outcomes for patients, especially at the social level (2–5). The phenomenology of anhedonia and motivational impairment appear similar in depression and schizophrenia (SZ), but likely arise through different underlying neural mechanisms. In depression, reduced reward responsivity may largely account for anhedonia and motivational impairment, whereas in SZ these behaviors may originate from deficits in neural correlates of cognitive control for effort-based decision-making (6). Despite the considerable prevalence of anhedonia and its related constructs in many psychiatric indications, few studies have evaluated these symptoms as a primary focus of drug development [e.g., see Lally et al. (7)], as their resolution was assumed to occur with successful treatment of the core indication (8).

In an effort to improve drug development and discovery in this important area of unmet need, modern behavioral neuroscience has sought to understand anhedonia and negative symptoms as trans-diagnostic deficits in reward processing in the context of the Research Domain Criteria (RDoC) framework (9–11). Brain imaging and electrophysiology methods such as functional magnetic resonance imaging (fMRI) and electroencephalography (EEG) have been used to develop behavioral reward processing tests. These tests provide objective and quantifiable measures of aspects of anhedonia and impaired motivation, which may represent drug-sensitive functional biomarkers for cross-disorder domains of dysfunction. These tests include paradigms probing anticipation and consumption of positive stimuli, delay discounting, reward response bias, prediction error (the difference between the expected and actual outcome of an action), reinforcement learning, and willingness to exert effort to acquire rewards. While some tasks require simultaneous assessment of brain activity using methods such as fMRI [e.g., in the Monetary Incentive Delay task (12)] or EEG, others (including the Effort Expenditure for Rewards Task [EEfRT; Treadway et al. (13)] can be used as standalone measures, facilitating their use in larger clinical studies.

Combining behavioral methods with neuroimaging offers an opportunity to identify targetable neurobiological biomarkers associated with different reward processing abnormalities. In drug development, reward processing tasks may be used to evaluate early pharmacodynamic activity in smaller translational studies; however, such tasks are rarely included in large, multisite clinical trials across different diagnoses and/or countries. It is still debatable whether they are sufficiently standardized, sensitive, and reliable for use in either go/no-go decisions or as endpoints in late-stage drug development studies. While the reliability of these measures has been extensively studied, there has been no systematic validation of the behavioral, fMRI, and EEG paradigms used to evaluate aspects of reward processing across the many tasks available (14). Reward processing tasks are typically implemented in single-site academic studies with small sample sizes, and it is common for task parameters to vary either subtly or substantially between research studies. These differences make comparisons of results or pooling of data across studies problematic. In addition, robust data on test–retest reliability is scarce, and often specific to one task in the context of a single indication, further limiting generalizability to other experimental settings. Standardized and systematically optimized tasks are required to leverage their full potential in drug development.

Despite the trans-diagnostic manifestation of reward processing deficits, prior studies have typically focused on differences between one particular diagnostic group and healthy controls, and rarely include multiple populations with different central nervous system disorders. Exploring transdiagnostic samples should help to determine whether seemingly similar deficits arise from disparate mechanisms in different disorders (6). This type of approach, which has been successfully implemented in early-phase drug development programs to treat anhedonia across diagnoses [e.g., the NIMH FAST-FAIL initiative, Krystal et al. (15)], could help to optimize reward processing paradigms for use in clinical trials. Furthermore, the relationship between behavioral endpoints and the severity of anhedonia and impaired motivation is poorly understood for any given indication.

Few studies have evaluated the sensitivity of these tasks to pharmacological interventions. Amphetamine-induced improvements in EEfRT performance have been demonstrated in healthy individuals (16), and the 5-HT reuptake inhibitor escitalopram can increase motivation to exert effort for reward in the Grip Strength Effort task (17). In individuals with Parkinson's disease, dopamine depletion reduces motivation as measured by the Grip Strength Effort task (18), and dopamine replacement can restore cognitive motivation (19). Other studies have shown that pharmacological manipulation of dopaminergic signaling in healthy individuals can influence choice performance in instrumental learning tasks (20, 21).

Currently available research rarely includes multiple tasks assessing different subdomains of reward processing. Assessing different subdomains of reward processing in the same participants would help to understand the contribution of the different components and subdomains (6). Multiple paradigms are required to assess reward valuation, reward responsiveness and reward learning separately (14, 22), but these have been largely studied in isolation rather than in a complementary manner. Studies comparing multiple paradigms that address the same reward processing sub-construct are also valuable, but remain scarce. One such study (23, 24) compared five effort-based decision-making paradigms related to reward valuation for use in clinical trials of individuals with SZ. Each paradigm was evaluated on its ability to differentiate patients and healthy controls, test–retest reliability, utility as a repeated measure (practice effects), patient tolerability, and correlations with self-reported and clinically rated motivational disturbances. The five paradigms showed varying psychometric strengths and weaknesses, with the EEfRT (13) and Grip Strength Effort task (25, 26) deemed most promising for use in clinical trials subject to some modifications and refinements (24).

However, these studies do not offer a complete trans-diagnostic picture of reward processing tasks and their potential usefulness as endpoints in clinical trials. Rather than repeating the same experiments across different positive valence subdomains and indications, we aim to integrate findings from available literature and optimize the most promising of these tasks for implementation in future large-scale, multisite clinical trials. The culmination of this process would be a first step toward a neuroscience-informed reward processing test battery, similar to those used to assess cognitive impairments, which could objectively quantify anhedonia and differentiate between impaired subdomains in different psychiatric disorders. The Reward Task Optimization Consortium (RTOC), supported and fostered by the European College of Neuropsychopharmacology Experimental Medicine Framework, was formed to facilitate this development process.

The first consortium study aims to conduct initial work toward the optimization of three experimental tasks, to support operational deployment of these tasks as endpoints in interventional clinical trials. The task battery comprises the Grip Strength Effort task (24), the Doors task (27), and the Reinforcement Learning/Working Memory (RLWM) task (28), evaluating reward responsiveness, reward valuation and reward learning, respectively. These tasks were selected by consensus based on a comprehensive review of publications demonstrating specificity for subdomains, maturity of the tasks for use in trials, accessibility, construct validity, and tolerability (14, 23, 24).

Our study objectives comprise:

1. Develop software and hardware to implement the task battery in clinical trials compliant with regulatory standards, while evaluating and maximizing suitability for multisite implementation in different languages.
2. Define a standardized set of task parameters.
3. Confirm operational deployment in multisite studies by ensuring that task outcome measures are broadly comparable to previously published data.
4. Replicate previously reported differences in performance and neural activity of participants with MDD or SZ compared with healthy controls, where available.
5. Evaluate test–retest reliability for key task outcome measures in a subset of participants. Good test–retest reliability and utility as repeated measures are necessary characteristics for tasks used as endpoints in clinical trials (29, 30), and would further confirm the suitability of our task battery for deployment in clinical trials.

In addition, we also plan to compare and contrast the pattern of reward processing deficits (assessed with the three proposed tasks) observed in the MDD and SZ groups, and to assess the relationships between behavioral/EEG data and clinical interview/self-report measures of anhedonia.

## Methods

### Design

All participants will complete a study visit comprising screening, administration of three reward processing tasks, and completion of a short set of clinical assessments and questionnaires measuring anhedonia/motivational deficits/negative symptoms.

Initially, 8 healthy control participants will be recruited to address any clearly evident implementation issues regarding hardware or software use, and to ensure that mean performance on behavioral tasks and EEG read-outs are broadly in alignment with expectations based on the published literature. Subsequently, individuals with MDD or SZ will be recruited until 37 participants per group have completed the study. Further healthy control participants will be recruited, for a maximum of 80 healthy control participants, to ensure acceptable age and gender matching between healthy control and patient groups.

To investigate test–retest reliability, 16 participants from each group will be recruited to attend a further retest visit 3–5 weeks after the initial visit. The retest visit will comprise re-administration of the task battery as well as a subset of the clinical assessments of anhedonia and negative symptoms. Patients and control groups participating in the retest visit will not be matched, but we will ensure each retest group is broadly representative of its own initial group in terms of age and gender.

The study commenced in September 2019 and is expected to run until the end of Q4 2020, accounting for 3 months of downtime due to the COVID-19 pandemic and resulting Europe-wide lockdowns.

### Participants and Procedure

Subject recruitment and study visits will take place at four European research centers: Maastricht University, University Hospital Frankfurt, Institute of Neuropsychiatry and Addictions Parc de Salut Mar of Barcelona, and Aristotle University of Thessaloniki. Each center will aim to recruit similar numbers of patients and controls (10 individuals with SZ, 10 individuals with MDD, and 20 healthy controls) and similar numbers of retest participants (4 individuals with SZ, 4 individuals with MDD, and 4 healthy controls). Participants will be 20–55 years of age.

Patients will partly be identified through clinical programs affiliated with the participating research sites, during clinical assessments. Healthy control participants and individuals with MDD or SZ will also be recruited through social media and advertising, and from cohort registers of patients who have agreed to be informed about future studies. Participants will be contacted using their preferred mode of communication. Eligibility of potential participants based on the major study criteria will be assessed via a short telephone screening. Eligible participants will receive the participant information sheet and will be offered adequate time to consider whether or not to participate. Where eligible participants indicate they would like to participate, the study team will help them plan their study visit.

Eligibility criteria are listed in Table 1. At the retest visit, participants will be asked about their recent medical history and medication use to assess their continued eligibility. Baseline data for participants excluded from retest will be included in analyses as long as the eligibility criteria were met at baseline.

**Table 1:** Eligibility criteria.

**Group**	**Inclusion criteria**	**Exclusion criteria**	**Retest visit exclusion criteria**
All participants	Age of 20–55 years	Diagnosis of schizoaffective disorder, bipolar disorder, eating disorder, obsessive-compulsive disorder, post-traumatic stress disorder, attention deficit hyperactivity disorder or autism spectrum disorder (healthy controls and individuals with MDD are also excluded if diagnosed with any psychotic disorder)	Use of opioids, psychostimulants or benzodiazepines within 2 weeks, or diazepam within 4 weeks prior to retest
		History of substance use disorder according to DSM-5 criteria, except nicotine or caffeine, within 6 months prior to screening	In the opinion of the investigator, too ill to participate or likely to present a danger to themselves or others
		Diagnosis of any clinically significant neurological disease (e.g., multiple sclerosis, epilepsy), mental retardation, or developmental disability	
		Use of opioids, psychostimulants or benzodiazepines within 2 weeks, or diazepam within 4 weeks prior to screening	
		In the opinion of the investigator, too ill to participate or likely to present a danger to themselves or others	
Individuals with MDD	Primary DSM-5 diagnosis of MDD, confirmed by the result of the MINI conducted by the site at screening		
	Current major depressive episode according to DSM-5 criteria, which has not lasted longer than 6 months		
	If undergoing treatment, treatment with an antidepressant for at least 4 continuous weeks		
Individuals with SZ	Primary DSM-5 diagnosis of SZ, confirmed by the MINI at screening	Treatment with clozapine in the last 6 months before screening	Treatment with clozapine between screening and retest
		Acute exacerbation requiring hospitalization within the last 3 months	
		Score >3 (mild) in Clinical Global Impression of Parkinsonism as measured by ESRS-A at screening	

*DSM-5, Diagnostic and Statistical Manual of Mental Disorders 5th edition; ESRS-A, Extrapyramidal Symptom Rating Scale–Abbreviated; MDD, major depressive disorder; MINI, Mini-International Neuropsychiatric Interview; SZ, schizophrenia*.

At the initial study visit, participants who have provided informed consent will be screened by trained researchers using the MINI for axis I disorders (31), the Quick Inventory of Depressive Symptomatology – Self-Report (QIDS-SR-16) (32), the Positive and Negative Symptom Scale (PANSS; individuals with SZ only) (33), the Extrapyramidal Symptom Rating Scale–Abbreviated (ESRS-A; individuals with SZ only) (34), and a comprehensive questionnaire capturing demographics, relevant medical history, and current disease management. Eligible participants will complete the selected anhedonia scales and the three reward processing tasks, in the following order: Grip Strength Effort task, Doors task, RLWM task. The Grip Strength Effort and Doors tasks will be administered in conjunction with EEG recording. The session, including EEG setup, instructions between individual tasks, and refreshment breaks, will last approximately 165 min. At the end of the study visit, participants will be reimbursed for their participation; participants will be informed that the amount of reimbursement will depend on task performance, although in reality all participants will receive a fixed amount. Participants also attending the retest visit will be reimbursed separately after completion of each visit.

Participants who withdraw or fail to complete all study procedures will be replaced until 37 participants per group have completed the initial visit. If, in the opinion of the investigator, the EEG data obtained for a participant is not suitable for analysis, the participant will be replaced.

### Reward Processing Paradigms

#### Task Selection

The three tasks selected for optimization in this study were chosen based on a review of the published literature, which demonstrates that these tasks are advanced in terms of methodological aspects and/or are suited to measure a specific RDoC domain/subdomain of reward processing. The evidence to support the selection each of these tasks is discussed below.

The Grip Strength Effort task measures reward valuation based on participants' willingness to exert effort for variable levels of reward (24, 35) with good reliability (intra-class correlation coefficient: 0.63), tolerability, and patient/control discriminant validity (23, 24). Moreover, the Grip Strength Effort task has a straightforward experimental design that easily lends itself to concurrent measurement of brain activity using EEG, a novel combination that will be used in the present study to investigate the spatiotemporal neural dynamics of reward anticipation and effort cost in the context of cost-benefit decision-making.

The Doors task is the RDoC paradigm of choice for measuring initial responsiveness to positive (gain) and negative (loss) outcomes, in conjunction with neurophysiological assessments to measure mesocorticolimbic activity during reward processing (36–38). Reward-related responses to this task have often been quantified in terms of feedback negativity, an event-related potential (ERP) index of reward evaluation (36). The feedback negativity ERP is positively correlated with behavioral and self-report measures of the participant's sensitivity to reward (38) and is reduced in individuals experiencing depressive symptoms (27, 37), suggesting good construct and discriminatory validity.

The RLWM task (28) was selected to isolate the contribution of working memory processes to reinforcement learning, which are demonstrated by the effects of load and delay (39, 40), and thus enables a more specific assessment of reward learning in this study.

The present study aims to optimize these three tasks for computerized, in-clinic administration with minimal participant burden across research centers in four European countries and their respective languages, in agreement with the stringent data integrity and standardization requirements for use in drug development studies.

We will employ several measures to ensure standardized operational deployment of the task battery. We will conduct standardized face-to-face training of key staff and supply standardized manuals to help maintain consistent high standards of data collection across all study sites, and hold regular all-site meetings to discuss operational performance. Test runs of behavioral and EEG data collection will also be conducted prior to the study start, to identify potential issues and check the data for abnormalities. The task battery will be delivered to all study participants at the initial and retest visits across all sites using the P1vital® ePRO system (P1vital Ltd, UK), a readily scalable online platform. A standardized script agreed by staff at all sites and translated into the relevant languages (Dutch, Spanish, German, and Greek) will be used to provide instructions to the participants, who will then complete standard practice trials for each task. However, while the key elements of the task delivery will be standardized, the exact presentation of the tasks may be adapted to fit site-specific conventions, such as the way in which monetary amounts are displayed (e.g., €0,40 or 0.40€).

#### The Grip Strength Effort Task

**Figure 1:**
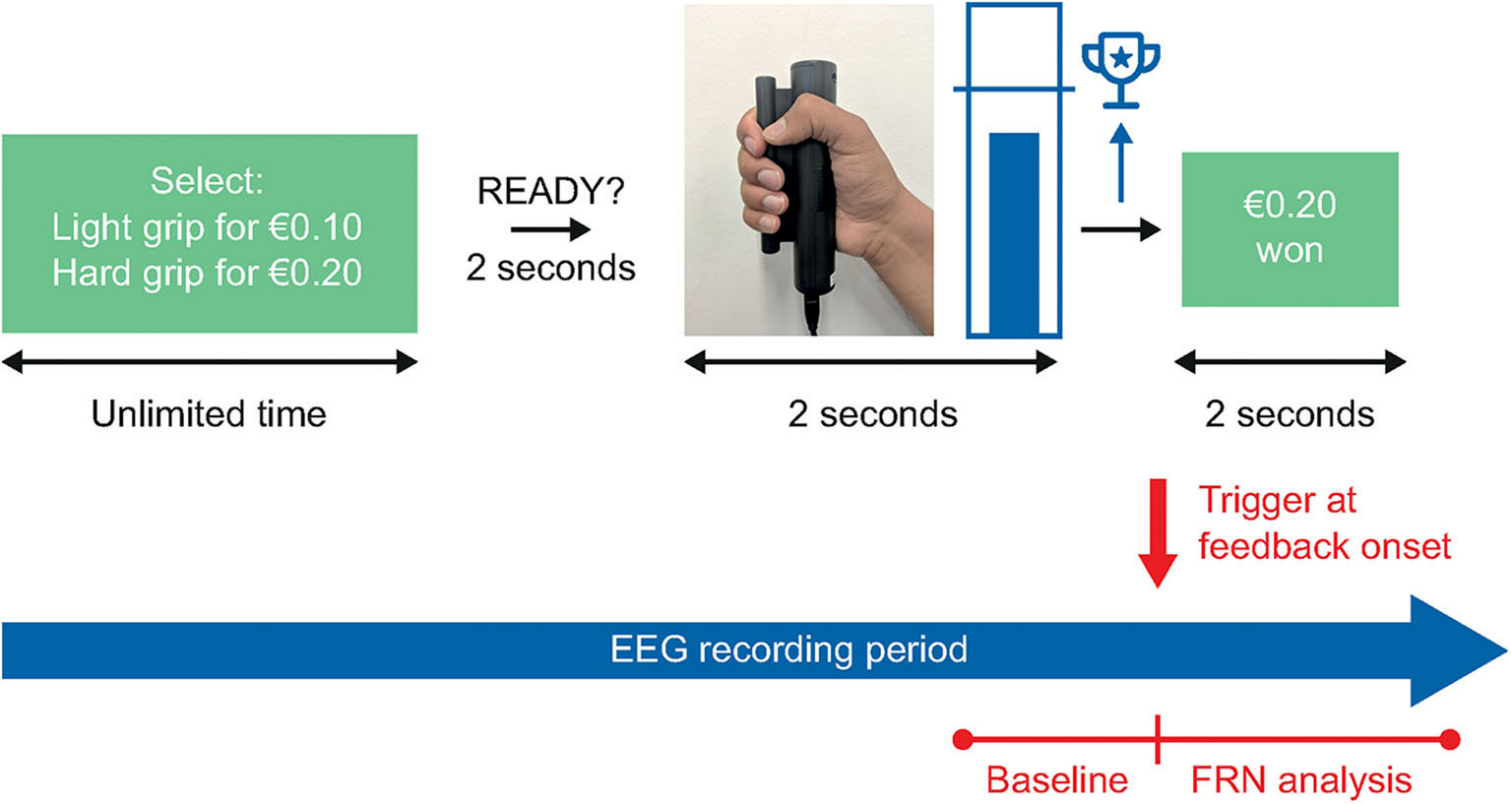
The Grip Strength Effort task. In the Grip Strength Effort task, participants will complete 4 practice trials, 4 calibration trials, and 54 assessment trials, for a total task duration of approximately 20 min. EEG, electroencephalography; FRN, feedback-related negativity.

The Grip Strength Effort task (Figure 1) measures participants' willingness to exert effort for variable monetary rewards, a proxy measure for motivation. On each trial, participants choose to perform either an easy or hard grip. The hard grip requires the participant to squeeze a grip force response device at a high percentage of their maximum possible grip strength (e.g., 90%), while the easy grip requires a lesser force (e.g., 50% of their maximum). The easy grip is always worth a specific lower amount (e.g., €0.10), while the reward associated with the hard trial varies (e.g., between three levels of reward value, €0.10, €0.20, or €0.40). The primary dependent variable is the percentage of hard grip choices at the different reward levels.

During a trial, participants receive visual feedback of the force produced via a thermometer scale marked with a target line, representing the target grip strength. If the participant reaches the target grip strength, they receive the reward associated with their grip strength choice on that particular trial. The task is preceded by a calibration step, which allows for the targets and visual feedback to be tailored to the participant's performance.

Participants complete a series of four practice trials prior to the assessment task. Two of these practice trials present a forced choice to allow the participant to experience both trial types; on the first the participant can only choose the easy task, and on the second they can only choose the hard task. The subsequent two practice trials are identical to the main assessment, in that participants can freely choose either the easy or hard task. The practice trials can be repeated as many times as needed to learn the task, and no performance criterion is required to proceed to the main assessment.

We are not aware of any published studies combining the Grip Strength Effort task with EEG recording, but the straightforward task design readily lends itself to capturing brain activity in the present study.

#### The Doors Task

**Figure 2:**
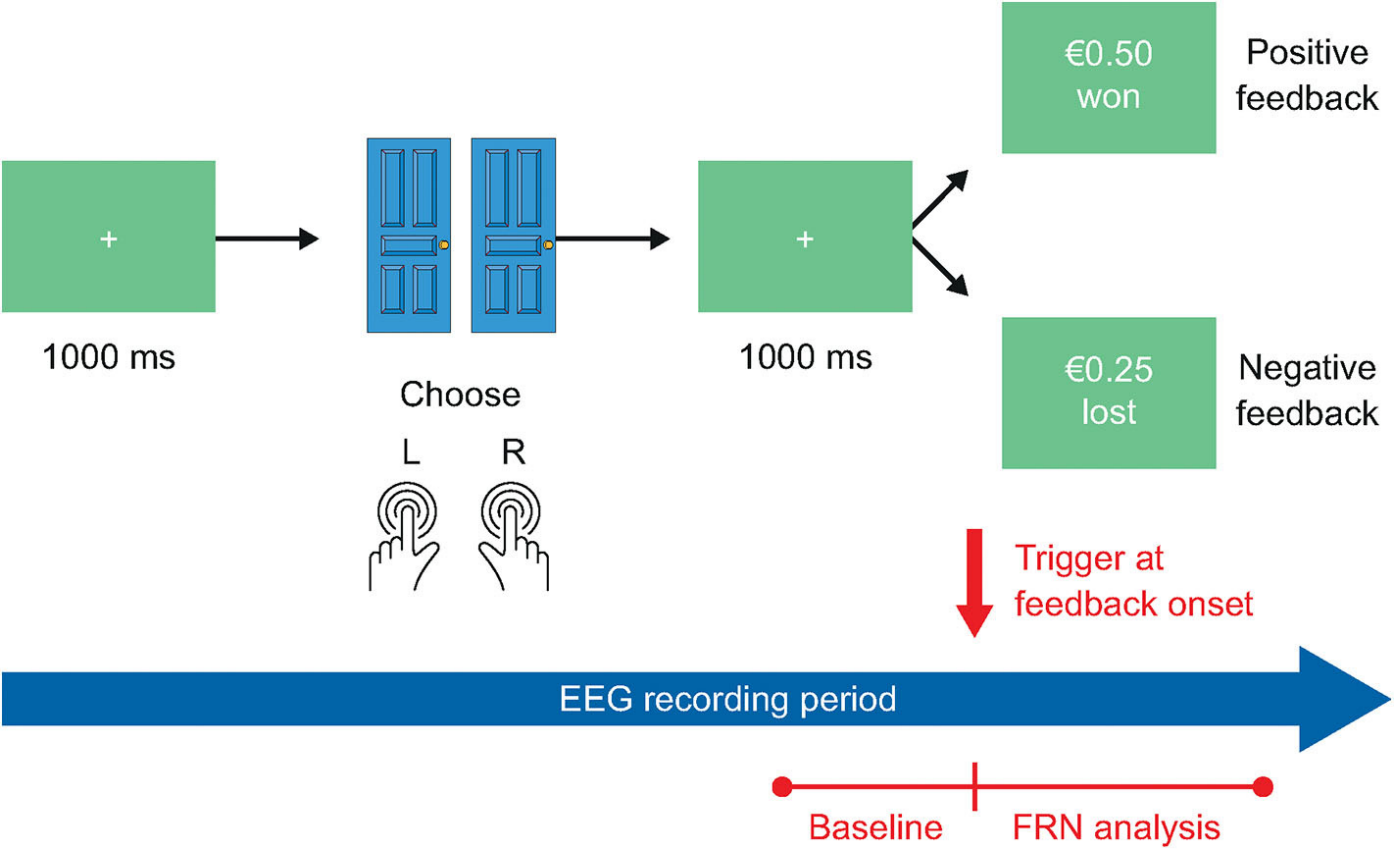
The Doors task. In the Doors task, participants will complete 4 practice trials and 60 assessment trials, for a total task duration of approximately 15 min. EEG, electroencephalography; FRN, feedback-related negativity.

The Doors task (Figure 2) measures initial responsiveness to unpredictable reward receipt and loss and must be paired with brain imaging measures that can quantify this construct, such as fMRI or EEG. On each trial, participants choose between two identical stimuli (often two doors) presented on the left and right side of the screen. Wins and losses are presented in a pre-determined, pseudorandom order, such that left or right door choices do not influence results and participants achieve exactly 50% wins associated with a positive reward (e.g., +€0.50) and 50% losses (e.g., −€0.25). The Doors task will be used in conjunction with EEG recording in the current study, as described in previous studies.

Participants will complete four practice trials prior to the main assessment, in which the participant can choose freely between the two doors, and feedback is presented as described above. The practice trials can be repeated as many times as needed to learn the task, and no performance criterion is required to proceed to the main assessment.

#### The RLWM Task

**Figure 3:**
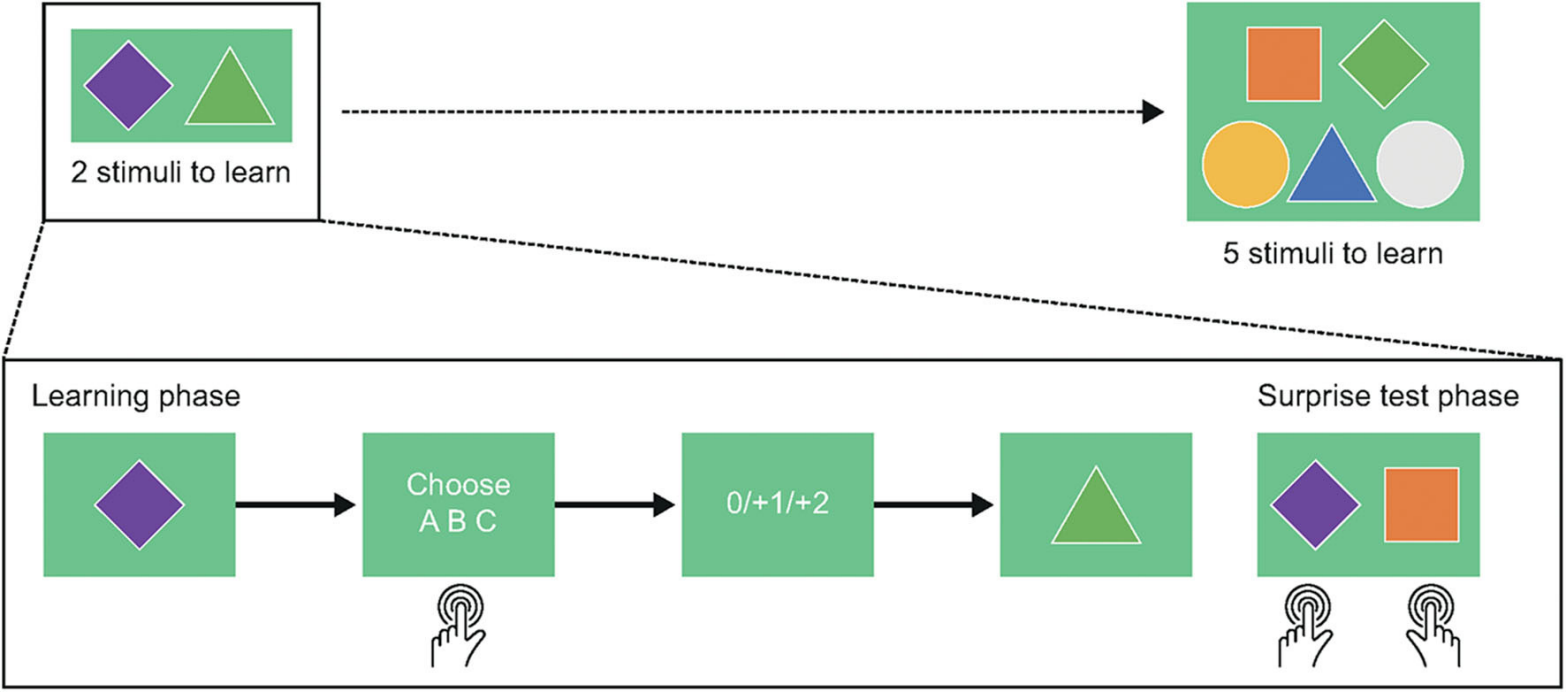
The Reinforcement Learning/Working Memory task. In the Reinforcement Learning/Working Memory task, participants will complete 43 practice trials, 507 learning trials, and 156 test trials, for a total task duration of approximately 40 min.

The RLWM task (Figure 3) is a new experimental protocol developed over multiple iterations by Collins and co-workers (28), which measures participants' ability to learn reward-based associations, and separately identifies the contributions of reinforcement learning and working memory to performance.

Firstly, participants learn to select one of three actions (e.g., key presses) for each stimulus in a block using reward feedback (learning phase). Incorrect choices provide no reward (0 points), while correct choices lead to reward (either +1 or +2 points, probabilistically). The probability of obtaining 2 points vs. 1 point is fixed for each stimulus and is drawn from a set of 0.2, 0.5, or 0.8 (reinforcement learning manipulation). Stimuli are presented individually, randomly intermixed, and the number of stimuli in a block varies from 2 to 5 (working memory manipulation).

Following the learning blocks, participants are presented with an unexpected test of RL-based contributions to learning (transfer phase). On each test trial, participants choose which of two stimuli previously encountered in the learning blocks is more rewarding. No feedback is provided during this phase, so performance is entirely based on the participant having learned the probabilistic reward magnitude associations for all stimuli, which might depend on the size of the block the stimulus was drawn from.

Prior to the main assessment, participants will complete 43 practice trials across two blocks related to the learning phase. In the first block comprising four trials, participants learn to associate shapes with rewards without any time restrictions, allowing the researcher to coach the participant through the completion of the task. In the second block, responses must be submitted within a 3 s time limit, and a missed response is recorded if the time limit is exceeded. This time limit is also enforced in the main assessment. In the second half of this block, participants must achieve 65% correct responses; if this criterion is not met, the practice trials must be repeated. If three unsuccessful attempts are made, the researcher is instructed not to proceed any further with the task. No practice trials are provided for the transfer phase, to preserve the surprise element.

### Self-Report and Clinical Assessments of Anhedonia and Related Constructs

Participants will complete several self-report and clinical assessments in addition to those included in screening. These include two self-report measures of anhedonia/motivation, the Snaith-Hamilton Pleasure Scale (SHAPS) (41) and the Behavioral Inhibition System/Behavioral Approach System scale (BIS/BAS) (42), as well as the Brief Negative Symptom Scale (BNSS) (43). These measures were selected based on short length and/or ease of administration, good construct validity and good reliability. All patient-reported scales will be delivered at the initial and retest visits using the P1vital® ePRO system.

#### SHAPS

The self-report, 14-item SHAPS was developed to measure hedonic capacity (41). Participants report levels of consummatory anhedonia over the last few days on a 4-point scale from strongly agree to strongly disagree. The SHAPS will be administered to all study participants.

The SHAPS is commonly used to assess anhedonia in depressed and suicidal populations (44–46), and has excellent reliability (Cronbach's alpha coefficient >0.9), satisfactory construct validity, and particularly promising convergent validity (44, 47).

#### BIS/BAS

The self-report BIS/BAS is comprised of 24 items in four subscales: BIS, BAS drive, BAS reward responsiveness, and BAS fun-seeking. The BAS subscales are designed to measure motivation toward a desired goal or rewarding stimulus, whereas the BIS subscale assesses the participant's desire to avoid an unpleasant situation or outcome (42, 48). Each item is scored from 1 (strongly agree) to 4 (strongly disagree). The BIS/BAS will be administered to all study participants.

The BIS/BAS has been used in studies of mood disorders including bipolar disorder and depression, and in SZ (49–52). The reliability of the scale has been found to be favorable in a large sample of individuals with anxiety and mood disorders (Raykov's rho: 0.88) (49).

#### BNSS

The BNSS is a researcher- or clinician-administered 13-item scale, which assesses five domains of negative symptomatology in SZ: alogia, anhedonia, avolition, asociality, and blunted affect. Each item is rated from 0 (non-existent) to 6 (highest severity) (43). The BNSS is almost exclusively administered to participants with SZ and SZ-type disorders (53–55) and will only be administered to individuals with SZ in the present study.

The BNSS has been found by two separate studies to have excellent reliability, with Cronbach's alpha coefficients >0.9 (43, 56), and also has good convergent and discriminant validity (57).

### EEG Data Acquisition

To control for variability in EEG recording conditions, an EEG kit, including EEG caps and a specific EEG acquisition system (amplifier and dedicated laptop) will be provided by Biotrial Inc. to each clinical site. At each site, the same EEG kit will be used for the whole set of tests performed during this study. EEG will be recorded on 64 channels, with a sampling frequency of 2,048 Hz and resolution of 16 bits. Raw EEG data will be recorded with a 0.1–70 Hz filter. A digital narrow 50-Hz centered notch filter will be applied to the data to reduce electromagnetic noise due to main power supply. Self-calibration and impedance measurement modules are integrated in the acquisition system.

EEG signals will be collected using EEG caps with electrodes located according to the International 10–20 Jasper system, and conductive gel will be used to ensure good contact between electrodes and the scalp, and to reduce impedance. In order to improve the artifact rejection process, two electrooculography (EOG) channels will be recorded using electrocardiogram patch electrodes. Vertical and horizontal EOG are bipolar channels that detect movements of the eyes and eyelids. Artifacts including high frequency noise due to muscular activity, environmental noise (i.e., electric peak or wire oscillation), and eye or eyelid movements (i.e., blinks) will be rejected by visual inspection.

## Statistical Analyses

The primary objective of the study is to evaluate operational deployment of the behavioral tasks by replication of published results, as determined by statistically significant separation of the respective indication groups from the healthy controls on the primary task variable for each of the three tasks.

The sample size was calculated using G^*^Power 3.1.9.2 (58) and powered to detect a statistically significant main effect of group on the percentage hard trials chosen in the Grip Strength Effort task, with a significance threshold of *p* < 0.05, power of 0.95, Cohen's *f* = 0.3516, and three measurements (low, medium, high levels of reward in the Grip Strength Effort task). Cohen's f was calculated based on the effect size estimate (partial eta^2^) reported by a previous study that employed a probabilistic reward learning task (59) to measure similar reward-related underlying psychological constructs to the tasks employed in the current study. The required sample was calculated as *N* = 37 per group; the study will therefore aim to recruit 40 participants per patient group, to accommodate a modest degree of drop-out or non-compliance.

After the first 8 healthy control participants have completed their study visit, we will conduct an initial analysis to address any evident implementation issues regarding hardware or software use. These analyses will comprise evaluations of: participants' understanding of the tasks, especially where there is a choice to be made; biological plausibility of response times; task duration; and amount of reward earned. Where possible, we will also assess whether primary behavioral and EEG task endpoints are in alignment with the published literature. The 8 subjects included in the initial analysis will not be included in the main analyses.

Primary and secondary task endpoints will be compared between patient groups and their age and gender-matched controls (two-way group design of MDD and matched controls and also SZ and matched controls). No additional correction for multiple comparisons is planned. Efficacy outcomes for each task will be described by age, gender, site and level of education. These variables will also be adjusted for the case-control comparison, if possible. We will also control for the effects of medication class, given our inclusion criteria permit the participation of medicated patients with SZ and MDD. Prior to database lock and analysis, data on participant medication will be classed according to compound and dose, in consultation with a study psychiatrist. Typical classes for this sample are expected to include atypical antipsychotics, sedatives, SSRIs and other mood stabilizers. A three-way group comparison between the SZ, MDD, and control groups may also be performed.

The study efficacy outcomes will be descriptively summarized by site to explore potential cultural differences that are known to be associated with response. Sites will be considered surrogates to cultural differences since they represent differences in location, language, and potentially in ethnicity.

Further analyses will include anhedonia/motivational scores, as measured by the SHAPS, BNSS, and BIS/BAS, as covariates. This approach will also allow investigation of the influence of anhedonia (and subdomains) on task performance across all participants, regardless of their indication. Furthermore, interactions between clinical assessment scores and group will indicate where effects of anhedonia may be specific to patient group(s).

Test–retest reliability for the three reward processing tasks will be evaluated separately for each participant group using intraclass correlations. Variability of selected task endpoint scores for the same participant across the two time points will be compared with the total variation across all endpoint scores for all participants within each group.

Participants with missing and/or unusable data for any of the three tasks will be excluded from that particular analysis. Where self-report data is missing and/or incomplete, the participant's behavioral data endpoints will still be included in the analysis where possible.

Computational modeling approaches may also be applied to yield further insights. This approach has been exemplified by an exploratory re-analysis of a study assessing effortful goal-directed behavior (60).

### Individual Paradigm Analyses

#### Doors Task

Performance in the Doors task will be analyzed as described previously (61) in terms of modeling the reward feedback stage of the task.

Stimulus-locked ERPs will be averaged separately for losses and gains, using the 200 ms period before stimulus onset as baseline. The primary task endpoint will be the feedback-related negativity (FRN) component of the ERP, defined as the activity on losses minus the activity on gains occurring at the FCz electrode site approximately 300 ms following feedback delivery. The difference score measures neural sensitivity to outcome valence regardless of the source, and this scoring method has been used in a number of prior studies (27, 62–69). Feedback-locked FRN will be averaged separately for each feedback type (i.e., gain, loss) and will be scored as the most negative peak occurring around 300 ms after feedback onset, using a 200 ms period prior to feedback onset as baseline.

The relative effect sizes of other secondary biomarkers, comprising reward positivity (RewP), frontal alpha (8–13 Hz) asymmetry, spectral analysis, and P300 will also be compared. RewP will be quantified as the mean activity in a specified time period after gain feedback onset at FCz. The P300 peak will be defined by its delay following feedback onset, amplitude (μV), latency (msec) and area under the curve (μV^*^msec). Spectral analysis of absolute and/or relative amplitude will be performed in delta, theta, alpha, beta and gamma bands, as defined by the guidelines for the recording and evaluation of pharmaco-EEG data in human participants (70).

#### Grip Strength Effort Task

The primary endpoint for this task will be the percentage of hard grip choices at each reward level (€0.10, €0.20, and €0.40). FRN will be the main reward feedback EEG biomarker of interest, and RewP and frontal asymmetry be investigated in a secondary analysis.

Feedback-locked FRN will be averaged separately for each level of reward value (€0.10, €0.20, and €0.40) and will be scored as the most negative peak occurring 200–400 ms after feedback onset. A 200 ms period prior to feedback onset will be used as baseline. The level of difficulty of the task (easy vs. difficult) will also be considered, to control for cases where the same level of reward (€0.10) could correspond to either a low-effort/low-reward or high-effort/low-reward option. While the Grip Strength Effort task is well-established and has the properties required for generating ERPs at the time of feedback, no electrophysiologically coupled studies that could guide our analyses have been conducted so far. However, based on existing fMRI studies, we assume a certain degree of similarity in electrophysiological responses between the Doors and the Grip Strength Effort tasks.

#### RLWM Task

The RLWM task will be analyzed as described previously (28). This approach includes investigation of differences in overall percentage of correct responses in the learning phase and percentage of correct responses in each block size between the patient and healthy control groups.

In order to quantify the effects of working memory and reinforcement learning on task performance, we will also conduct trial-by-trial logistic regression analyses on data from both the learning and transfer phases. For the learning phase, we will model individual participants' choice of response on each trial as a function of the following predictor variables: set size (number of stimulus images in the block), iteration (how many times the stimulus has been encountered), pcor (number of previous correct choices for the current stimulus), and delay (number of trials since the last correct choice for the current trial's stimulus). We will then compare the effects of each of these predictor variables on learning across participant groups.

For the transfer phase, we will define the following characteristics for each stimulus: value (reward history; the average of all feedback received for this image), set size, and block (number of the block in which the stimulus image was encountered). Logistic regression modeling of individual participants' choices will be used to quantify the effect of these characteristics on performance in the transfer phase, and these effects will be compared between groups.

## Discussion

This pilot study will evaluate and optimize the operational deployment of three reward processing tasks for use in future large-scale, international clinical trials. While these tasks are extensively used in neuropsychiatric research, they have not been developed to meet the regulatory standards required for use in pharmaceutical industry-sponsored trials, and the task parameters can vary substantially between studies. By replicating previous findings in two patient populations and healthy controls in a tightly controlled experimental setting, we will provide initial evidence of the trans-diagnostic reliability and test–retest consistency of our chosen tasks to support their use in future validation studies. This trial began recruiting in November 2019, and completion is anticipated in Q4 2020.

The present study will also add to the growing body of literature supporting the identification of reliable biomarkers for cross-disorder domains of dysfunction, and explore differences in reward processing deficits, as measured by the test battery, in individuals with MDD and SZ compared with matched controls. Previous research has demonstrated that both MDD and SZ are associated with decreased willingness to expend effort, blunted striatal responses during reward anticipation, and reduced prediction error signaling to rewards relative to healthy controls (71). Although it has been argued that the mechanisms of reward circuit dysregulation underlying anhedonia and impaired motivation may differ between the two diagnoses (72), these conclusions have often been based on literature reviews and meta-analyses of studies using disparate methodologies. The interpretation of results across studies using conceptually similar paradigms can be challenging due to differences in the specific methodology, such as differing task reward values or the use of probabilistic vs. deterministic reward reinforcement (6). Few studies have employed the same tasks to investigate reward processing in a trans-diagnostic manner [see Arrondo et al. (73) for an example of a study across multiple indications], and, to our knowledge, no previous study has employed a battery of tasks targeting different reward processing constructs in multiple patient groups compared with healthy controls. As reward processing deficits are multifaceted rather than the result of a disturbance to a singular process, our study design might be better placed to provide initial evidence for where deficits are similar and where they diverge across different patient populations.

Notably, the current study is the result of a pre-competitive collaboration between academic researchers and representatives from the pharmaceutical industry. This approach to achieving common aims and objectives has become increasingly popular, due in part to the economy of scale that pre-competitive consortia can provide (74). Moreover, bringing together industry experts who have extensive experience with experimental medicine models in psychiatry and academics at the forefront of neuropsychiatric research ensures that the projects conducted within the pre-competitive consortium framework remain relevant to drug development while also exploring innovative methodologies to fulfill ambitious objectives. As such, the pre-competitive consortium platform established for this study provides a promising framework for the future development, optimization, and operationalization of the selected reward processing tasks and additional anhedonia biomarkers.

Certain limitations in the design of this study should be noted, which present research questions to be addressed in future studies. Potential order effects due to the consistent task order in all patients (Grip Strength Effort task, Doors task, RLWM task) could vary across clinical populations. However, the consistent task order in the present study may be appropriate given our focus on exploring operational feasibility for interventional studies, where assessments typically follow a fixed task schedule to avoid confounding of the primary comparison between intervention and control. The present study also places limited restrictions on medication use among participants, although all participants receiving treatment are required to be on a stable medication regimen, raising the possibility of varying treatment effects between participants receiving different medications. Future research exploring the sensitivity of these paradigms to medication use is needed to support validation of their use in interventional clinical trials; however, the present study may permit exploration of residual symptoms of anhedonia and its behavioral correlates in a cross-sectional sample of patients, many of whom will be well-managed and receiving effective medication. Finally, this study did not incorporate cognitive testing to identify any baseline differences in cognitive ability between cohorts, although the RLWM task will be used to assess how working memory impacts reward learning performance across the patient and control groups.

In conclusion, the proposed study takes the first step toward optimizing a neuroscience-informed battery of experimental tasks assessing different facets of reward processing for use in future large clinical trials across multiple disease indications.

## Ethics Statement

This study has been granted ethical approval by a local Ethics Review Board at each of the four study sites, including the Medical Ethical Committee of Maastricht University, who will act as study sponsor. The trial will be conducted in compliance with the principles of the 1996 Declaration of Helsinki, the Medical Research Involving Human Subjects Act (WMO), and Good Clinical Practice and in accordance with all applicable regulatory requirements including, but not limited to, the Research Governance Framework. Written informed consent will be obtained from all study participants.

## Author Contributions

GRD and SP conceived of the research study and provided key input for the devising the protocol, the writing of which was coordinated by AB. ARa prepared the first draft of the manuscript. DH, AH, DU, DP, JT, AS, TA, ARe, GP, VP, ME, DM, VB-A, and BS contributed to the protocol design and review of the final manuscript. DM and VB-A provided expertise for EEG data collection and analysis. DH provided expertise for the Reinforcement Learning/Working Memory task design and analysis. All authors contributed to the article and approved the submitted version.

## Conflict of Interest

The authors declare that this study received funding from Boehringer Ingelheim International GmbH, H. Lundbeck, Janssen Pharmaceutica, BlackThorn Therapeutics, and F. Hoffmann-La Roche Ltd. All members of the consortium, including representatives from the funders, provided their expertise to ensure the optimal study design, contributed to preparation of the manuscript, and were involved in the decision to submit for publication. AB, ARa, AH, and GRD are employees of P1vital Ltd. DU is a full-time employee of F. Hoffmann-La Roche. DP is a full-time employee of Janssen Pharmaceutica. JT is a full-time employee of Blackthorn Therapeutics. BS is a full-time employee of Lundbeck. DM and VB-A are full-time employees of Biotrial Neuroscience. VP has been a consultant to or has received honoraria or grants from AstraZeneca, Bristol-Myers Squibb, Janssen Cilag, Lundbeck, Otsuka, Servier, Medtronic, and Exeltis. SP is a full-time employee of Boehringer Ingelheim. The remaining authors declare that the research was conducted in the absence of any commercial or financial relationships that could be construed as a potential conflict of interest.
